# Urine Metabolomic Patterns to Discriminate the Burnout Levels and Night-Shift-Related Stress in Healthcare Professionals

**DOI:** 10.3390/metabo15040273

**Published:** 2025-04-14

**Authors:** Andreea Petra Ungur, Andreea-Iulia Socaciu, Maria Barsan, Armand Gabriel Rajnoveanu, Razvan Ionut, Carmen Socaciu, Lucia Maria Procopciuc

**Affiliations:** 1 Department of Occupational Health, Iuliu Hatieganu University of Medicine and Pharmacy, Str. Victor Babes 8, 400347 Cluj-Napoca, Romania; andreea.ladaru@umfcluj.ro (A.P.U.); maria.opritoiu@umfcluj.ro (M.B.); armand.rajnoveanu@umfcluj.ro (A.G.R.); ionut.razvan@umfcluj.ro (R.I.); 2 Research Center for Applied Biotechnology and Molecular Therapy BIODIATECH, SC Proplanta Str. Trifoiului 12G, 400478 Cluj-Napoca, Romania; csocaciu@proplanta.ro; 3 Department of Molecular Sciences, Medical Biochemistry, Iuliu Hatieganu University of Medicine and Pharmacy, 400012 Cluj-Napoca, Romania; lprocopciuc@umfcluj.ro

**Keywords:** burnout syndrome in healthcare workers, night shift work, urinary metabolomics, emotional exhaustion, low personal accomplishment, depersonalization, biomarkers, metabolic pathways

## Abstract

Burnout syndrome, which significantly impacts both individual and societal quality of life, is primarily characterized by three key criteria: depersonalization, emotional exhaustion, and low personal accomplishment, all linked to work-related stress. **Purpose**: Comparative evaluation of urine metabolite patterns that may discriminate the burnout levels and the effects of night shifts on healthcare professionals. The Maslach Burnout Inventory survey was administered to 64 physicians and nurses working day and night shifts, with scores for each criterion recorded. **Methods**: Urine samples were collected, and metabolomic patterns were analyzed using UHPLC-QTOF-ESI+-MS technology. This analysis employed both untargeted and semi-targeted metabolomics, coupled with multivariate and ANOVA statistics, utilizing the online Metaboanalyst 6.0 platform. Partial Least Squares Discriminant Analysis (PLSDA) was performed, along with VIP values, Random Forest graphs, and heatmaps based on 79 identified metabolites. These were further complemented by biomarker analysis (AUC ranking) and pathway analysis of metabolic networks. **Results**: The findings highlighted the biochemical effects of night shifts and their correlation with burnout scores from each dimension. **Conclusions**: This study demonstrated the involvement of three major metabolic pathways in diagnosing burnout: lipid metabolism, particularly related to steroid hormones (cortisol, cortisone, and androsterone metabolites); energetic metabolism, involving long-chain acylated carnitines as transporters of free fatty acids, which play a role in burnout control; and a third pathway affecting catecholamine metabolism (neurotransmitters derived from tyrosine, such as dopamine, adrenaline, and noradrenaline), as well as tryptophan metabolism (serotonin and melatonin metabolites) and amino acid metabolism (including aspartate, arginine, and valine).

## 1. Introduction

Burnout syndrome is defined as a stress-related condition with a range of signs and individual symptoms, often linked to various metabolic changes, prolonged fatigue, and a sense of frustration or dissatisfaction [1,2,3,4]. According to the International Classification of Diseases (ICD-11), it is defined by emotional, physical, and mental exhaustion. In occupational medicine, burnout has been associated with several risk factors, including night shifts and work overload, which have been shown to increase the risk of cardiovascular diseases, psychiatric disorders, and certain types of cancer [5,6,7,8,9]. The burnout phenomenon is work-related and can occur regardless of the profession, often overlapping with anxiety and depression, as highlighted by recent research [10,11,12,13,14,15,16]. It is typically characterized by three dimensions: depersonalization (DP), emotional exhaustion (EE), and low personal accomplishment (PA). The impact of burnout is commonly assessed using the Maslach Burnout Inventory (MBI) questionnaire [17], which is widely utilized to measure the effects of occupational stress resulting from work overload and poor job management, leading to chronic stress. Medical personnel are particularly vulnerable to burnout [18,19,20,21,22], which is why a specific version of the MBI has become the most validated tool for assessing burnout in healthcare workers. The data that we obtained from this questionnaire have been correlated with various risk factors [23] as well as endocrinological and immunological markers of burnout, which are involved in specific metabolic pathways [24,25,26,27,28,29,30].

Occupational burnout syndrome has been linked to insomnia and metabolic dysfunctions, with recent studies exploring biological parameters and sleep disturbances in relation to burnout symptoms among workers [2,4]. Experimental studies have found significant correlations between burnout and circadian dysfunction, particularly in individuals who work night shifts, showing alterations in plasma or urinary concentrations of melatonin, a signaling molecule known as a “chronobiotic hormone” or “dark hormone”. Tryptophan, the precursor to serotonin, is enzymatically converted into melatonin, a process influenced by beta-adrenergic receptors. The study of melatonin, along with its precursors and metabolites, has gained significant international scientific interest, as they are correlated with multiple pathological processes, including cardiovascular, neurological, and cancer-related diseases, as well as important endocrinological and metabolic alterations [4,31,32,33,34,35,36]. Since melatonin’s release is regulated by both diurnal and nocturnal metabolic processes and follows a circadian rhythm in response to natural light changes, its levels in the blood and urine remain of particular interest, especially among healthcare professionals working night shifts. Additionally, variations in the blood levels of certain hormones, like cortisol and dehydroepiandrosterone sulfate, have proven useful in assessing prolonged exposure to stress, with changes influenced by circadian rhythms [37,38,39,40,41]. A study comparing participants with burnout to healthy individuals in terms of professional rank, sleep, job strain (measured by the Karasek questionnaire), social support, anxiety, and depression (assessed using the HAD scale) found that higher concentrations of glycemia and glycosylated hemoglobin, along with elevated levels of cholesterol, triglycerides, C-reactive protein, and thyroid-stimulating hormone, as well as lower levels of 25-hydroxyvitamin D, could serve as predictive factors for burnout. In contrast, models incorporating job strain, job satisfaction, anxiety, and insomnia did not effectively predict burnout [2]. Various advanced techniques for analysis, ranging from enzyme immunoassays (ELISA) to radioimmunoassay, and particularly high-performance liquid chromatography coupled with mass spectrometry, have demonstrated their ability to deliver precise results by enabling the separation and identification of numerous metabolites, within the broader framework of high-throughput metabolomics technology. Within metabolomics, both untargeted and targeted approaches have been used to study stress-related metabolites, with recent research primarily focusing on serum and salivary neurotransmitters, as well as hormones like cortisol, ACTH, and prolactin [21,42,43,44]. However, fewer studies have examined urine metabolites [45,46,47] and their correlation with blood biomarkers [7].

There have been limited reports on burnout syndrome in Romania [4,26], especially among healthcare professionals. Our previous investigations [4,36,48] utilizing metabolomics approaches concentrated on blood biomarkers for medical professionals categorized by the Maslach Burnout Inventory (MBI) (based on the three criteria: DP, EE, and PA) and associated with circadian dysfunction due to night shifts. This study aims to provide new insights into burnout biomarkers in urine samples collected from the same subjects and compare them with previously obtained blood metabolomics data. The untargeted and semi-targeted approaches enabled a more comprehensive understanding of common and specific biomarkers involved in metabolic pathways related to burnout, highlighting the relevance of urine as a biofluid for accurately reflecting burnout levels.

## 2. Materials and Methods

### 2.1. Patients and Study Design

This study adhered to the guidelines outlined in the Declaration of Helsinki and the Conference for Coordination of Clinical Practice, and it was approved by the Ethics Committee for Scientific Research (DEP231/21.07.2023) of the “Iuliu Hațieganu” University of Medicine and Pharmacy, Cluj-Napoca, Romania. Written informed consent was obtained from all participants. A total of 64 medical care professionals (doctors and nurses) from hospitals in the Transylvania region of Romania participated in this study and completed the Maslach Burnout Inventory-Human Services Survey (MBI-HSS) during September and October 2023. The responses were collected through direct interviews. Blood and urine samples were collected from these participants, with blood analysis data recently published [48].

As mentioned previously, burnout levels were assessed using the Maslach Burnout Inventory (MBI), licensed from Mind Garden. The MBI questionnaire consists of 22 items: emotional exhaustion (EE) was evaluated through questions 1, 2, 3, 6, 8, 13, 14, 16, and 20; depersonalization (DP) was assessed through questions 5, 10, 11, 15, and 22; and personal accomplishment (PA) was evaluated through questions 4, 7, 9, 12, 17, 18, 19, and 21. Scoring was performed according to the MBI interpretation key. Participants were classified based on their work conditions (night/day shifts) and according to the three MBI criteria (DP, PA, and EE) into high-burnout (H) (in this category we included high and middle scores) and low-burnout (L) (in this category we included low scores) groups, as detailed in Table 1.

Blood samples were collected from all subjects. At the time of blood sampling, the participants were also provided with urine collection containers and instructions on how to properly collect the samples. However, not all of the participants returned their urine samples, which may explain the higher number of blood samples compared to urine samples (*n* = 97 blood vs. *n* = 64 urine).

The inclusion criteria were as follows: complete and accurate responses to all questionnaires, signed consent to participate in the study, consent for both blood and urine sampling, active medical personnel (doctors, nurses, healthcare workers) with at least 6 months of professional experience in healthcare, exposure to both day and night shifts for comparison, and no known conditions that could interfere with the study (e.g., neurological or psychiatric disorders). Additionally, the participants needed to have regular work schedules involving factors related to burnout.

The exclusion criteria included employees from sectors outside of healthcare, participants who did not provide informed consent, and those who refused to provide biological samples.

### 2.2. Sample Preparation

Urine samples were collected the day after the interview, in the morning around 6 a.m., before breakfast, in sterile vials, according to thorough previous instructions. They were preserved by adding 0.1% Na azide and stored at −80 °C until analysis, labeled with confidential numerical codes. For each 0.2 mL of urine, 0.8 mL of a mix of HPLC-grade methanol and acetonitrile (2:1 *v*/*v*) was added. The mixture was vortexed to precipitate proteins, ultrasonicated for 5 min, and stored at −20 °C for 24 h to enhance the protein precipitation. After centrifugation at 12,500 rpm for 10 min at 4 °C, the supernatant was collected and filtered through 0.25 μm nylon filters. The resulting solution was placed in glass micro-vials and introduced into the autosampler of the ultra-high-performance liquid chromatograph (UHPLC) before injection. Quality control (QC) samples were prepared by mixing 0.2 mL of urine from each sample and running at the start and after every 10 samples to verify the reproducibility of the LC-MS analysis.

### 2.3. UHPLC-QTOF-ESI+-MS Analysis

Metabolomic profiling was carried out using ultra-high-performance liquid chromatography coupled with electrospray ionization quadrupole time-of-flight mass spectrometry (UHPLC-QTOF-ESI+-MS) on a Thermo Fisher Scientific UHPLC Ultimate 3000 system, equipped with a quaternary pump, Dionex delivery system, and MS detection system with MaXis Impact (Bruker Daltonics, Berlin, Germany). Metabolites were separated on an Acclaim C18 column (5 μm, 150 mm x 2.1 mm, 30 nm pore size, Thermo Fisher Scientific, Waltham, MA, USA) maintained at 28 °C. The mobile phase consisted of 0.1% formic acid in water (A) and 0.1% formic acid in acetonitrile (B) (Li-Chrosolv^®^ Merck Millipore, Darmstadt, Germany). The gradient program and MS parameters were previously outlined [48]. A 5 µL volume of the extracted sample was injected, with the column set at 25 °C. The MS parameters included positive ionization mode (ESI+), MS calibration with sodium formate, a capillary voltage of 3500 V, nebulizing gas pressure set to 2.8 Bar, a drying gas flow rate of 12 L/min, and a drying temperature of 300 °C. The *m*/*z* range for separation was set between 100 and 800 Daltons. Instrument control and data processing were performed using TofControl 3.2, HyStar 3.2, Data Analysis 4.2 (Bruker Daltonics), and Chromeleon software version 7.2.

### 2.4. Statistical Analysis

By UHPLC-QTOF-ESI+-MS analysis, approximately 850 molecules were separated from the urine samples. The acquired data were processed using Data Analysis 4.2 software. Initially, the TIC (Total Ion Chromatogram) and BPC (Base Peak Chromatogram) were generated using specific algorithms. Following this, a comprehensive matrix that included all samples was created, using the methodology applied previously for blood serum samples [48]. The advanced bucket matrix was generated using the Find Molecular Features (FMF) algorithm, which included details for each *m*/*z* value, retention time, peak area, and peak intensity.

For identification and statistical analysis, a series of filtering steps were performed. First, MS peaks with retention times under 0.8 min, intensities below 3000 units, S/N values less than 10, and *m*/*z* values above 800 Daltons were excluded. In the second step, alignment of the *m*/*z* values was performed using the online tool at www.bioinformatica.isa.cnr.it/NEAPOLIS (accessed on 15 November 2024), with the common molecules found in over 60% of the samples retained. After these procedures, 79 molecules were identified and selected for further metabolomic analysis, which was performed using the Metaboanalyst 6.0 platform (https://www.metaboanalyst.ca/MetaboAnalyst, accessed on 12 January 2025) for both multivariate and univariate analyses.

The significant metabolic patterns were identified by comparing the experimental *m*/*z* values with theoretical *m*/*z* values from established international databases, such as the Human Metabolome Database (HMDB), LipidMaps, and PubChem. The comparison of these values showed an accuracy of below 20 ppm between the theoretical and experimental *m*/*z* values.

In the initial phase of the statistical analysis, the focus was on performing untargeted, multivariate analysis of the detected molecules from group 1 (night shift workers) versus group 0 (control group). Additionally, for each criterion (DP, EE, and PA), the low burnout level (L) was compared to the high burnout level (H), as outlined earlier. Supervised discriminations between these groups were determined using Partial Least Squares Discriminant Analysis (PLSDA) and Random Forest (RF)-based prediction tests, and visualized through heatmap clusters and correlations. The Variable Importance in the Projection (VIP) values and graphs of RF Mean Decrease Accuracy (MDA) were calculated to rank the most significant molecules responsible for group discrimination.

Further biomarker analysis was conducted using ROC curves, and the Area Under the Curve (AUC) was determined as a complementary prediction method for potential biomarkers of differentiation. Finally, pathway enrichment analysis was applied to the identified cohort of 79 molecules to explore the relevant metabolic pathways involved.

The semi-targeted approach and statistical analysis were also applied, focusing separately on three classes of molecules: polar compounds (including neurotransmitters), steroids, and carnitines, which were identified as significant through the untargeted metabolomics approach. The putative biomarkers indicating metabolic disturbances induced by burnout were selected based on the literature data or filtered through the untargeted analysis.

To differentiate subjects based on day/night work and high/low burnout (H/L) for the three criteria (DP, EE, and PA), a one-way ANOVA was conducted, with Fisher’s post hoc LSD (Least Significant Difference) test applied for further comparisons. Finally, the Venny 2.1 algorithm (https://csbg.cnb.csic.es, accessed on 12 January 2025) was used to compare the data obtained from the urine metabolomic profile with the blood profile, identifying common or specific molecules as potential biomarkers.

## 3. Results

### 3.1. Stratification of Urine Samples According to Demographic Data and Burnout Scores

Table 2 presents details about the samples, including the type of work (day work or night shift work), gender distribution, and average burnout level scores. It also includes classification based on the MBI-HSS survey scores. The middle and high scores were combined and categorized as high (H), as the number of high scores was significantly lower than that of the middle scores.

**Figure 1:**
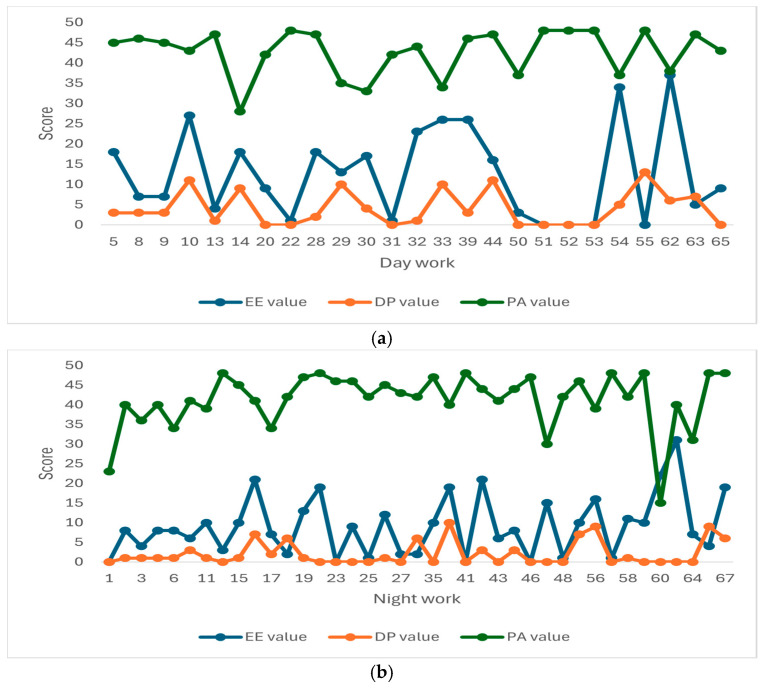
Individual values of emotional exhaustion (EE), depersonalization (DP), and personal accomplishment (PA) scores for each of the 64 patients. Patients were classified based on their work type: (**a**) 25 patients with day work, and (**b**) 39 patients with night work. According to the data presented in Table 2 and Figure 1a,b, in the night work group (**b**), positive correlations could be observed between the burnout scores and high burnout levels for the EE and DP criteria. The mean values for high burnout in EE and DP were 23.29 and 9.41, respectively, compared to 5.93 and 1.32 for low burnout, respectively.

The graphs presented in Figure 1 illustrate the individual distribution of burnout scores based on the criteria of depersonalization (DP), emotional exhaustion (EE), and personal accomplishment (PA). The thresholds for high (H) versus low (L) burnout were set at 16 for EE, 6 for DP, and 38 for PA. Graph (a) represents professionals with day work (*n* = 25), while graph (b) shows professionals with night work (*n* = 39).

### 3.2. Untargeted Metabolomic Profiles to Discriminate Metabolic Profiles Between Night Work and Day Work Subjects

**Figure 2:**
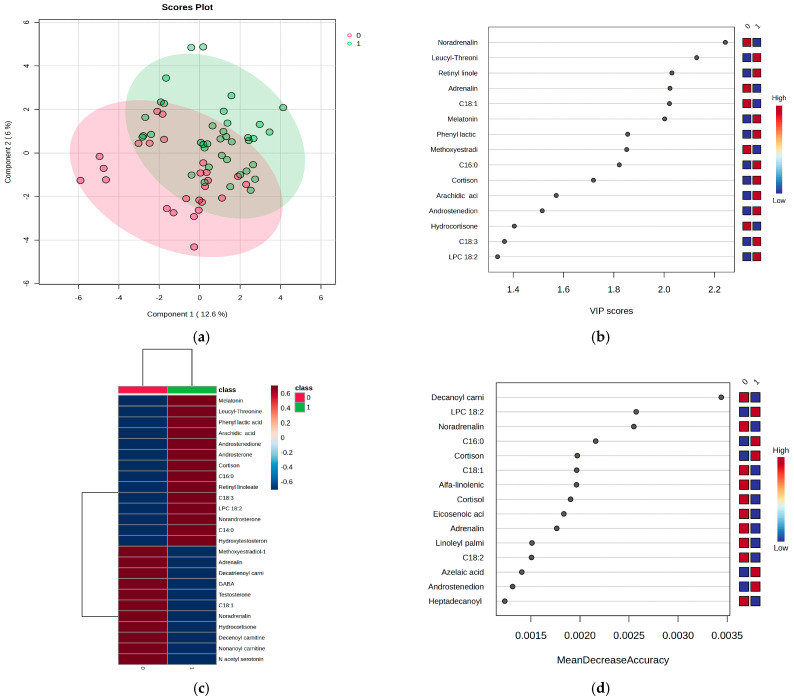
(**a**) PLSDA score plot, (**b**) VIP score graph, (**c**) heatmap representation, and (**d**) RF graph were generated to evaluate the metabolic differences between the two groups of subjects: day work (0) and night work (1). The PLSDA score plot (Figure 2a) reveals a covariance of 18.6%, with partial overlap between the two groups, but still showing an acceptable level of discrimination. The VIP scores (Figure 2b), which are above 2, highlight key findings in the night work group, including significant decreases in noradrenaline, adrenaline, and oleic acid (C18:1), along with increased levels of melatonin, leucyl-threonine, and retinyl linoleate. These findings are further confirmed by the heatmap representation (Figure 2c).

Based on the data obtained through UHPLC-TOF-ESI+-MS analysis, 79 metabolites were selected and identified, categorized into five molecular classes, as detailed in Supplementary File S1 (Table S1). Using multivariate statistical methods, including Partial Least Squares Discriminant Analysis (PLSDA), Variable Importance in the Projection (VIP) scores, heatmap visualization, and Random Forest (RF) analysis, the differences between the two groups of subjects (0—day work; 1—night work) were assessed for significance. The results of these analyses are displayed in Figure 2a–d.

The RF algorithm, with MDA values > 0.0020 (Figure 2d), revealed additional insights for the night work group, showing decreased levels of decanoylcarnitine, noradrenaline, and oleic acid, along with increased levels of LPC 18:2, palmitic acid, and cortisone. Moreover, the cortisone/cortisol ratio was examined in both groups based on LC-MS peak intensities. The night shift group exhibited an increase in the cortisone/cortisol ratio to 1.4, while metabolites such as hydrocortisone, dihydrocortisol, tetrahydrocortisone, and tetrahydrocortisol (as detailed in Figure S1) showed relatively constant ratios of around 1.0 when compared between the night and day work groups.

### 3.3. Biomarker and Pathway Analysis of Metabolites in Night vs. Day Work Groups

The Biomarker Analysis algorithm in Metaboanalyst 6.0 was used to generate Receiver Operating Characteristic (ROC) curves and determine the Area Under the Curve (AUC). These values provide insights into the sensitivity and specificity of potential biomarkers. The highest AUC values help rank the biomarkers that differentiate between the night work (1) and day work (0) groups. Table 3 presents the top 10 metabolites, including their AUC values greater than 0.60, *p*-values, Log2FC, and their respective changes in levels between the two groups (negative values when increased or positive values when decreased).

Based on our data, increased levels of urine melatonin, phenyl lactic acid, retinyl linoleate, leucyl-threonine, cortisone, and androstenedione were specific to the night work group. On the other hand, noradrenaline levels were decreased in this group.

**Figure 3:**
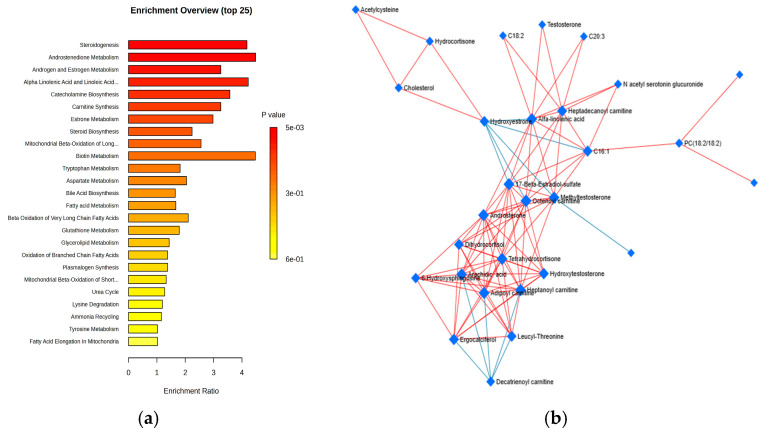
(**a**) Metabolic pathways influenced by night work in comparison to day work, identified through HMDB pathway enrichment analysis (with enrichment ratios up to 3.5), based on all of the molecules included in the statistical analysis. (**b**) DSPC network illustrating the molecular relationships within the metabolic pathways (from the KEGG pathway database).

Additionally, the cohort of 79 identified molecules underwent pathway enrichment analysis based on *p*-values and enrichment scores of up to 3.5, as shown in Figure 3a. Figure 3b presents the Debiased Sparse Partial Correlation (DSPC) network, which highlights the molecular relationships in the metabolic pathways (KEGG pathway database) related to the night shifts.

According to enrichment ratios, the most significantly affected pathways were steroid metabolism (particularly androstenedione, estrogen, and androgen metabolism), steroidogenesis, catecholamine biosynthesis (neurotransmitters derived from tyrosine, such as dopamine, adrenaline, and noradrenaline), and amino acid metabolism (involving tryptophan, aspartate, arginine, and valine), which also impacted carnitine synthesis and biotin (vitamin H) metabolism.

Additionally, the DSPC network of molecular interactions within metabolic pathways, derived from the KEGG pathway database, incorporates the metabolites and edges representing their associations, emphasizing the top 20% of correlations ranked by *p*-value. This network illustrates that burnout primarily involves lipid metabolism, particularly with hormonal effects (e.g., estrone, androstenedione, and cortisol derivatives), as well as lipophilic vitamins (such as retinol, tocopherol, and ergocalciferol) and acylated carnitines—key molecules involved in lipid transport into the mitochondria.

### 3.4. Untargeted Metabolomics Analysis of Burnout Criteria: DP vs. EE vs. PA for All Subjects

**Figure 4:**
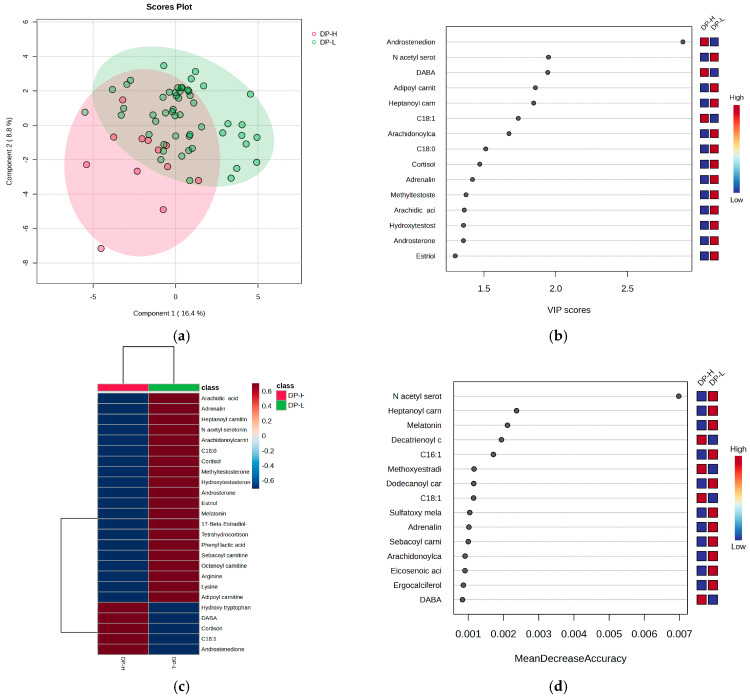
The (**a**) PLSDA score plot, (**b**) VIP graph, (**c**) heatmap, and (**d**) RF graph showing the most significant molecules that differentiate subjects based on the DP criterion for burnout.

**Figure 5:**
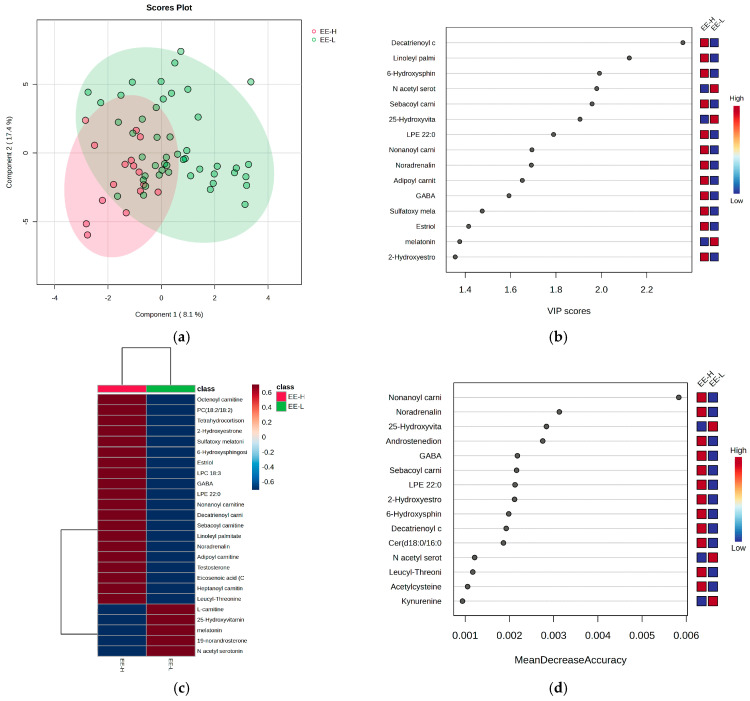
The (**a**) PLSDA score plot, (**b**) VIP graph, (**c**) heatmap, and (**d**) RF graph illustrating the most significant molecules that distinguish subjects based on the EE criterion for burnout.

**Figure 6:**
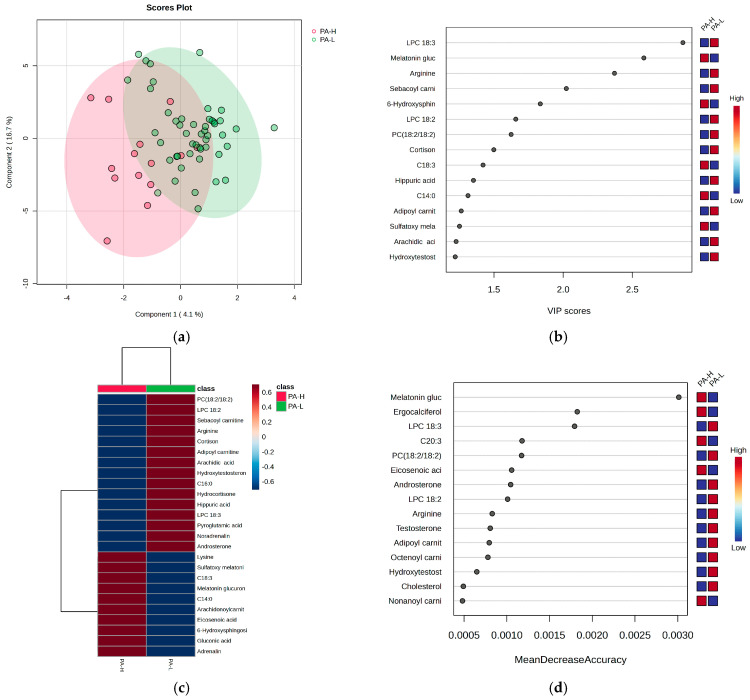
The (**a**) PLSDA score plot, (**b**) VIP graph, (**c**) heatmap, and (**d**) RF graph illustrating the most significant molecules that differentiate subjects based on the PA criterion for burnout. Considering the PA criterion, the PLSDA score plot (Figure 6a) shows a covariance of 20.8%. The VIP scores (Figure 6b), heatmap (Figure 6c), and RF graph with MDA values above 0.002 (Figure 6d) reveal elevated levels of melatonin glucuronide, hydroxy sphingosine, and ergocalciferol, along with sulfatoxymelatonin in the H group. Additionally, decreased levels of LC 18:3, arginine, and cortisone were observed in group H. No significant differences were noted for cortisol and acylated carnitines.

Considering the burnout criteria DP, EE, and PA for all subjects, regardless of their night or day work schedule, the PLSDA plots, VIP values, heatmaps, and RF graphs are displayed in Figure 4, Figure 5 and Figure 6, respectively. Figure 4 illustrates the statistical analysis that distinguishes subjects based on the DP criterion for burnout, highlighting the most significant molecules that contribute to the observed differences.

When considering the DP criterion, the PLSDA score plot (Figure 4a) demonstrates a covariance of 25.2%, with partial overlapping between the high- and low-burnout groups (H and L). Group L had a more homogeneous distribution compared to group H. The VIP scores (Figure 4b), heatmap (Figure 4c), and RF graph with MDA values greater than 0.002 (Figure 4d) indicate that the high-burnout group (H) had elevated levels of androstenedione, cortisone, DABA, and hydroxytryptophan, while showing decreased levels of N-acetyl serotonin, melatonin, cortisol, and long-chain acylated carnitines.

Figure 5 provides further statistics that reflect and differentiate the subjects according to the EE criterion for burnout, alongside the most significant molecules contributing to these differences.

Considering the EE criterion, the PLSDA score plot (Figure 5a) shows a covariance of 25.5%, with partial overlap between the H and L groups. The VIP scores (Figure 5b), heatmap (Figure 5c), and RF graph with MDA values above 0.002 (Figure 5d) reveal elevated levels of androstenedione, noradrenaline, GABA, and hydroxytryptophan in the H group, along with decreased levels of N-acetyl serotonin, melatonin, and hydroxyvitamin D. No significant differences were observed for cortisol and cortisone, while long-chain acylated carnitines were generally higher in the H group.

Figure 6 presents the statistics that distinguish subjects based on the PA criterion for burnout, highlighting the most significant molecules that may account for these differences.

### 3.5. Semi-Targeted Metabolomics Based on the Burnout Criteria DP vs. EE for the Three Classes of Molecules

From the cohort of molecules selected for statistical analysis, five classes were identified (polar metabolites, acylcarnitines, fatty acids and derivatives, steroid metabolites, and phospholipids), based on their involvement in specific metabolic pathways (see Supplementary File, Table S1).

Following the untargeted analysis, three classes of molecules were selected for their relevance in differentiating between night and day work: polar metabolites, including polar neurotransmitters, catecholamines, and amino acids (A); acylcarnitines (B); and steroids (C).

For each class, Metaboanalyst analysis was applied to the aligned matrices (*m*/*z* values vs. peak intensities), specifically considering the burnout criteria DP and EE to discriminate between the night work and day work groups.

**Figure 7:**
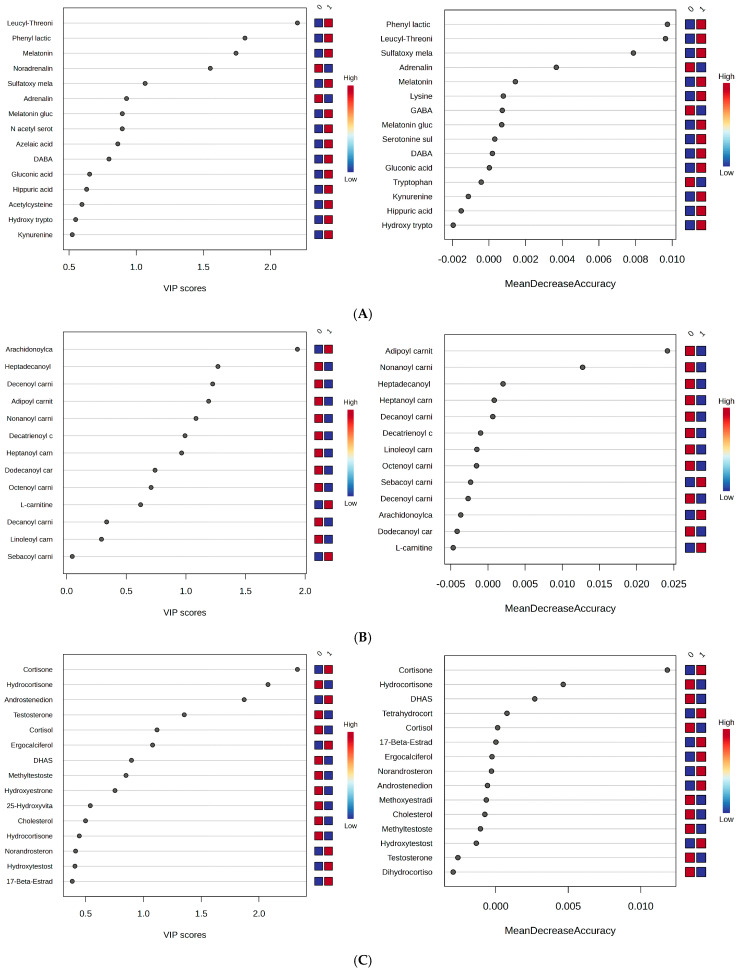
(**A**–**C**) VIP and RF graphs corresponding to each of the three metabolite classes (**A**–**C**) are provided to illustrate the differences between groups 0 (day work) and 1 (night shift): (**A**) Polar metabolites; (**B**) acylcarnitines; (**C**) steroid metabolites.

Figure 7A–C show the VIP scores and RF graphs to propose potential biomarkers that could differentiate the groups 0 (day work) and 1 (night shift).

Based on these results, specific variations across different classes of molecules were identified:

(1) Polar metabolites: Phenyl lactic acid, leucyl-threonine, melatonin, melatonin glucuronide, and sulfatoxymelatonin were found to be significant. Higher levels were observed for melatonin, melatonin glucuronide, and sulfatoxymelatonin in the night shift group, along with reduced levels of adrenaline and noradrenaline, GABA, and tryptophan at the time of urine collection.
(2) Long-chain carnitines (C16–C20): Apart from arachidonyl carnitine, these metabolites showed decreased levels in the night work group. Meanwhile, free carnitine was increased.
(3) Steroids: Cortisol and hydrocortisone, compared to cortisone, exhibited reverse relationships, with the former decreased in the night work group. A similar inverse relationship was observed between androstenedione and DHAS (a hormonal precursor of androgens and estrogens), where androstenedione levels were higher in the night work group. No significant changes were observed for estrone and testosterone metabolites.

### 3.6. One-Way ANOVA Statistics for Molecules Involved in DP, EE and PA Burnout Levels, Comparing Day Work vs. Night Work

Finally, ANOVA statistics were applied for an integrated approach, simultaneously comparing high (H) and low (L) burnout levels across all three criteria (DP, EE, and PA). The most significant graphs, including the RF graph and heatmaps, which highlight the putative biomarker molecules for discrimination, are presented in Supplementary File S2. Table 4 displays the comparative results of the ANOVA, using Fisher’s post hoc (Least Significant Difference) test.

In all cases, and especially when considering the DP criterion, variations were observed in the same cohort of molecules involved in burnout—specifically, melatonin and its metabolites (mainly sulfatoxymelatonin), androstenedione, and DHAS, as well as acylated carnitines. All previous statistics have confirmed these findings.

### 3.7. Comparative Analysis of Metabolite Findings in Urine Versus Blood Serum

Common and specific metabolites identified in urine were compared to similar studies conducted on blood serum [48]. The Venny 2.1 comparative diagrams highlighted the metabolites shared between blood and urine, as well as those found exclusively in either blood or urine, as detailed in Table 5.

## 4. Discussion

Research on stress dysfunctions and burnout has advanced significantly in recent years, with most studies conducted in the past three years, particularly small-scale occupational studies. These studies have utilized advanced techniques, such as metabolomics, to measure and evaluate various molecular biomarkers. Among the most notable biomarkers identified so far are cortisol, cortisone, and their metabolites (tetrahydrocortisol, dihydrocortisol, tetrahydrocortisone, etc.), which are found either free or conjugated as glucuronides in blood and urine, as determined by LC-MS [45,49]. Additionally, using LC-MS, melatonin and its precursors or metabolites, such as 6-sulfatoxymelatonin, have been identified in urine, reflecting the blood melatonin concentrations [46,50,51]. The urine metabolome has been corroborated with the plasma metabolome [7,42], revealing a prominent effect on tryptophan metabolism, marked by elevated levels of 3-indoxylsulfate (a tryptophan metabolite) and carnitine. In this context, this retrospective study focused on burnout syndrome among healthcare professionals working in clinics in the Transylvania region of Romania. The subjects were selected from various work environments (day work or exposure to circadian disruption through night shift work over a prolonged period). Burnout levels were assessed using the widely used MBI survey, and the resulting scores classified the subjects into High-burnout (H) and Low-burnout (L) categories based on three criteria: DP, EE, and PA.

Metabolomics studies in humans with circadian disturbances (such as those working night shifts) have shown changes in tryptophan metabolism (serotonin, N-acetylserotonin, melatonin, and its metabolites), neurotransmitters, steroids (mainly cortisol and its metabolites, androstenedione, and DHAS), and acylcarnitine metabolism.

Three main objectives were considered: the relationships between the metabolic profile for day versus night work (1); the correlations between different metabolites and burnout levels (H or L), based on the scores in each criterion (DP, EE, and PA) (2); and similar correlations for three classes of metabolites (3). The untargeted and semi-targeted metabolomics, coupled with multivariate statistics, revealed significant classes of metabolites that are interconnected within various metabolic pathway networks and correlated with burnout levels.

(1) The effect of night work on the metabolic profile, independent of burnout levels, was evaluated using untargeted metabolomics (Section 3.2 and Section 3.3). The VIP scores above 2, RF graphs, and heatmaps revealed significant changes in the night work group, including decreases in noradrenaline, adrenaline, decanoylcarnitine, and oleic acid (C18:1), as well as increased levels of melatonin, leucyl-threonine, retinyl linoleate, and cortisone. The cortisone-to-cortisol ratio in the night work group increased to 1.4, compared to day work, while their metabolites (hydrocortisone, dihydrocortisol, tetrahydrocortisone) maintained constant ratios around 1.0. According to biomarker analysis, AUC values > 0.6 indicated increased levels of melatonin, phenyl lactic acid, retinyl linoleate, leucyl-threonine, cortisone, and androstenedione in the night work group, while noradrenaline was reduced. Pathway analysis showed that the metabolic networks were significantly affected by night work compared to day work, as determined by HMDB pathway enrichment analysis and the DSPC network of molecular relationships in metabolic pathways (from the KEGG pathway database). The most impacted pathways included steroid metabolism (especially androsterone and its metabolites), catecholamine biosynthesis (neurotransmitters derived from tyrosine, such as dopamine, adrenaline, and noradrenaline), and tryptophan metabolism, which also influenced carnitine acylation. The DSPC network, based on intermolecular relationships, highlighted that burnout primarily involves lipid metabolism with hormonal impacts (e.g., estrone, androstenedione, and cortisol derivatives), as well as acylated carnitines, key molecules for lipid transport into the mitochondria.
(2) The untargeted metabolomics analysis considering the DP, EE, and PA criteria for all subjects (Section 3.4), independent of day or night work, showed differentiations in metabolites based on burnout levels (H or L). Under the DP criterion, increased levels of androstenedione, cortisone, DABA, and hydroxytryptophan, along with decreased levels of N-acetyl serotonin, melatonin, cortisol, and long-chain acylated carnitines, were observed in the H group compared to the L group. Under the EE criterion, increased levels of androstenedione, noradrenaline, GABA, and hydroxytryptophan were found, while N-acetyl serotonin, melatonin, and hydroxyvitamin D were decreased in the H group. No significant differences were observed for cortisol and cortisone, and long-chain acylated carnitines were generally higher in this group. Under the PA criterion, melatonin glucuronide, sulfatoxymelatonin, hydroxy sphingosine, and ergocalciferol showed increased levels, while arginine and cortisone were decreased in the H group. No significant differences were found for cortisol and acylated carnitines.
(3) A semi-targeted analysis was performed based on the previous untargeted metabolomics results (Section 3.5). From the entire cohort, molecules belonging to three metabolite classes were used to discriminate subjects with day vs. night work, considering the DP, EE, and PA criteria separately. These classes included polar metabolites (neurotransmitters, catecholamines, amino acids) (A), acylcarnitines (B), and steroids (C). The results showed that phenyl lactic acid, leucyl-threonine, melatonin, and sulfatoxymelatonin were upregulated in the night work group. Positive relationships were observed for melatonin and sulfatoxymelatonin, as well as for adrenaline and noradrenaline. Long-chain carnitines (C16–C20), except for arachidonyl carnitine, showed decreased levels in the night work group. Among the steroids, cortisol and hydrocortisone showed reverse relationships with cortisone, with the former being lower in the night work group. A similar reverse relationship was observed between androstenedione and DHAS (a hormonal precursor of androgens and estrogens), with increased androstenedione levels in the night work group. No significant changes were observed for estrone and testosterone metabolites.

Using ANOVA statistics (Section 3.6), an integrated approach was taken by selecting 21 metabolites that showed significant modifications in previous analyses (Section 3.2, Section 3.3, Section 3.4 and Section 3.5). These metabolites were compared for high (H) and low (L) burnout levels across the DP, EE, and PA criteria, as well as for day vs. night work. The top eight significant molecules across all criteria were adrenaline, melatonin, androstenedione, androsterone, DHAS, cortisol, and cortisone. The *p*-values were minimal for the DP criterion, indicating that this was the most significant criterion for discrimination, in comparison to the EE and PA criteria.

Finally, the results from this study were compared with metabolomics data from a previous study on blood sera from similar subjects. In the blood sera, 99 molecules were identified and characterized, while 79 molecules were identified in urine. Among them, 53 molecules were common to both biofluids, while 26 were found exclusively in urine and 46 exclusively in blood serum. The most representative molecules to be considered as putative biomarkers, based on all of the data presented, were adrenaline, noradrenaline, androsterone, norandrosterone, androstenedione, DHAS, melatonin, cortisol, cortisone, and long-chain acylated carnitines in both biofluids, while sulfatoxymelatonin and some cortisone metabolites were more specific to urine. Given these data, urine can be considered to be a very convenient biofluid for reflecting burnout levels and circadian disturbances.

## 5. Conclusions

The data obtained highlight the relevance of using urine as a convenient source of information that may reflect the biochemical implications of night shift work and burnout levels in various environments. Specifically, for healthcare professionals, notable metabolic changes were observed, correlated with burnout levels as determined by MBI scores.

In conclusion, three main metabolic pathways were identified as crucial in diagnosing burnout: lipid metabolism, particularly involving steroid hormones (such as cortisol, cortisone, and androsterone metabolites); energetic metabolism, with long-chain acylated carnitines acting as transporters of free fatty acids to help regulate burnout levels; and catecholamine metabolism (neurotransmitters derived from tyrosine, including dopamine, adrenaline, and noradrenaline), as well as tryptophan metabolism (serotonin and melatonin metabolites, aspartate, arginine, and valine).

Urine, in comparison with blood, offers valuable insights into the metabolic turnover and can be considered to be an effective and convenient biofluid for reflecting burnout levels and circadian disturbances.

## Figures and Tables

**Table 1:** Demographic information, type of work, and scores obtained by participants who provided urine samples were recorded, considering the three burnout criteria: DP (depersonalization), EE (emotional exhaustion), and PA (low professional accomplishment). Participants were categorized into high-burnout (H) or low-burnout (L) groups based on their scores.

	Number of Subjects	Day/Night Work	DP Scores	EE Scores	PA Scores
Total number	64	25/39	DP-L (0–6):52 81.2%	EE-L (4–16):47 73.4%	PA-H (15–38):14 21.8%
			DP-H (7–13):12 18.8%	EE-H (17–51):17 26.6%	PA-L (40–48):50 50%
F/M (number) F/M (mean age ± SD)	58 F/6 M 46.05 ± 8.2/50.8 ± 15.5	23 F/35 F 2 M/4 M			

**Table 2:** Details regarding the urine samples collected from medical care professionals, including the distribution of gender, age, and type of work (day/night shifts). The table also includes the burnout criteria scores for emotional exhaustion (EE), depersonalization (DP), and personal accomplishment (PA). The subjects are classified according to the thresholds of high (H) and low (L) burnout, as determined by the MBI-HSS survey scores.

Type of Work	Mean Scores ± SD		
	DP	EE	PA
Day (*n* = 25)	4.25 ± 4.3	13.25 ± 11.2	42.66 ± 5.82
Night (*n* = 39)	2.05 ± 2.9	8.92 ± 7.5	41.3 ± 7.05

**Table 3:** Ranking of the top 10 molecules, with AUC values > 0.600, to be considered putative biomarkers for differentiating between the night work group (group 1) and the day work group (group 0). Log2FC values and significance indicate whether the levels of these molecules have decreased (+) or increased (−) in group 1 compared to group 0.

Molecule	AUC	*p*	log2FC	Molecule	AUC	*p*	log2FC
Melatonin	0.653	0.105	−0.220	Androstenedione	0.619	0.201	−0.493
Phenyl lactic acid	0.639	0.081	−0.522	Arachidic acid	0.616	0.213	−0.109
Retinyl linoleate	0.631	0.117	−0.215	Cortisone	0.615	0.094	−0.605
Leucyl-threonine	0.622	0.064	−0.120	LPC 18:2	0.610	0.199	−0.584
Noradrenalin	0.622	0.041	0.310	C14:0	0.603	0.309	−0.305

**Table 4:** Comparative *p*-values determined from ANOVA using the 21 molecules found to have statistical significance in differentiating subjects with day vs. night work (0 vs. 1) and high (H) vs. low (L) burnout levels. (⇑)—increase; (⇓)—decrease.

DP-H (1) vs. DP-L (1) vs. DP-H (0) vs. DP-L (0)	*p*-Value	EE-H (1) vs. EE-L (1) vs. EE-H (0) vs. EE-L (0)	*p*-Value	PA-H (1) vs. PA-L (1) vs. PA-H (0) vs. PA-L (0)	*p*-Value
Adrenaline (⇓)	0.024	Melatonin (⇓)	0.118	DHAS	0.242
Androstenedione (⇑)	0.025	Sulfatoxymelatonin	0.213	Noradrenalin	0.255
Melatonin (⇓)	0.029	Androstenedione (⇑)	0.227	Androstenedione	0.298
Androsterone (⇑)	0.134	Cortisone (⇑)	0.290	Melatonin (⇓)	0.331
DHAS	0.176	Androsterone (⇑)	0.296	Hydrocortisone	0.358
Cortisol (⇑)	0.177	Noradrenalin (⇓)	0.298	Cortisone (⇑)	0.363
Cortisone (⇑)	0.180	Tetrahydrocortisone (⇑)	0.350	Hydrocortisone glucuronate	0.584
Tetrahydrocortisone (⇑)	0.191	Adrenalin (⇓)	0.459	Tetrahydrocortisone	0.604

**Table 5:** Common and specific metabolites found in urine versus blood serum, according to the metabolomics investigation. The molecules marked with * and bold were identified as statistically relevant and considered to be putative biomarkers.

53 Common for “Urine” and “Blood”	26 Exclusively Present in “Urine”	46 Exclusively Present in “Blood”
17-Beta-estradiol-sulfate	25-Hydroxyvitamin D3	(Iso)Leucine
2-Methoxyestrone	6-Hydroxysphingosine	11-Hydroxyandrosterone
Adrenaline *; noradrenaline *	Acetylcysteine	17-Methyltestosterone
Androsterone *; norandrosterone *; androstenedione *	Adipoyl carnitine	Estrone; 2-hydroxyestrone
Arginine; asparagine	Alpha-androstenol	2-Methoxyestradiol-17beta
C14:0; C16:0; C16:1	Arachidic acid	5 Hydroxy lysine; 5 OH tryptophan
C18:0; C18:1; C18:2; C18:3, C20:3	Arachidonoyl carnitine	5,6-trans-25-Hydroxyvitamin D3
Cortisol *; cortisone *; dihydrocortisol; tetrahydrocortisone	Azelaic acid	Acetyl-D-carnitine
DABA	Cer(d18:0/16:0)	C20:0; C20:1; C20:2; C20:4
DHAS *	Cholesterol	Ceramide(d18:0/16:0)
Ergocalciferol	Decatrienoyl carnitine	Dinor lithocholic acid
Estriol	Dihydrocortisone	Glucose; glutamine; methionine
GABA	Eicosenoic acid	Heptadecanoyl carnitine (C17:0)
Gluconic acid	Heptadecanoyl carnitine (C17:0)	Linoleoyl carnitine (C18:2)
Hippuric acid	Nonanoyl carnitine (C9:0)	Hydroxy glutamic acid
Kynurenine	Hydrocortisone glucuronide	LPC 18:0; LPC 18:1
L-carnitine	Hydroxy tryptophan	Methyl hippuric acid
Lysine; leucyl-threonine	Hydroxyestrone	Serotonin; N acetyl serotonin
Linoleyl palmitate	Linoleoyl carnitine	N methyl nicotinamide
LPC 18:2; LPC 18:3; LPE 22:0	Methoxyestradiol-17beta	N-acetyl spermidine
Melatonin *; melatonin glucuronide	Methyltestosterone	Octenoyl and palmitoyl carnitine
N acetyl serotonin glucuronide and sulfate	Palmitoyl and octenoyl carnitine	Palmitoleyl linolenate
PC (18:2/18:2)	Sebacoyl carnitine	PC (18:1/18:1); PC (18:1/18:2); PC (18:2/17:2)
Phenyl lactic and pyroglutamic acid	Serotonin sulfate	Tyrosine; proline; threonine; valine
Retinyl linoleate	Sulfatoxymelatonin *	Prostaglandin F2
Testosterone		Retinol (vitamin A)
Tryptophan		Sphingosine; 6-hydroxysphingosine
Haptanoyl, decanoyl, decenoyl, and dodecanoyl carnitine (C7:0, C10:0, C10:1, and C12:0)		Tocopherol; aspartic acid
18:0 Cholesterol ester		Tetrahydrocortisol
Hydroxytestosterone		

## Data Availability

All data are available in the manuscript.

## Supplementary Materials

The following supporting information can be downloaded at: https://www.mdpi.com/article/10.3390/metabo15040273/s1, Table S1. Urine molecules (*n* = 79) separated using HPLC-QTOF-ESI+-MS and identified according to their *m*/*z* values. The experimental *m*/*z* values were compared with the average of theoretical *m*/*z* values from the international database HMDB (Human Metabolomic Database); Figure S1. (a) RF graph and (b) heatmap illustrating the most representative molecules that may discriminate the EE-H vs. EE-L burnout scores of day (0) vs. night work (1) subjects.

## Abbreviations

The following abbreviations are used in this manuscript:

DP	Depersonalization
EE	Emotional exhaustion
PA	Low personal accomplishment
DHAS	Dehydroandrosterone sulfate
