# Supplementary material for: Urine Metabolomic Patterns to Discriminate the Burnout Levels and Night-Shift-Related Stress in Healthcare Professionals
Source: Metabolites. 2025 Apr 14;15(4):273. doi: 10.3390/metabo15040273 (PMC12029983; doi:10.3390/metabo15040273)
Supplement: Supplementary file 1 [file metabolites-15-00273-s001.zip › Figure S1.pdf]

**Supplementary file Figure S1.** Figure S2. RF-graph (a) and Heatmap (b) illustrating the most representative molecules which may discriminate the DP-H *vs* DP-L, EE-H *vs* EE-L and PA-H vs PA-L burnout scores of day (0) *vs* night work (1) subjects.

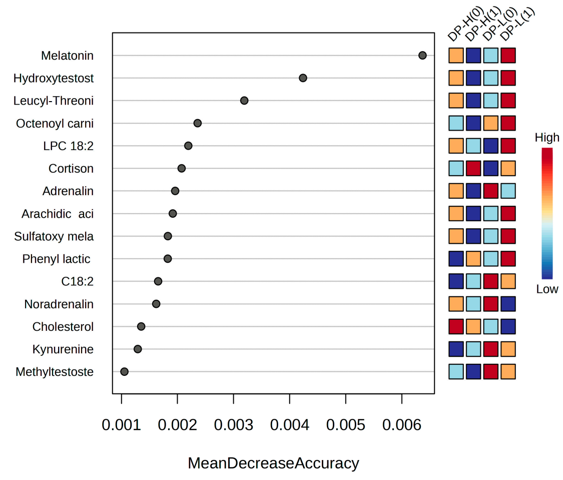


(a)

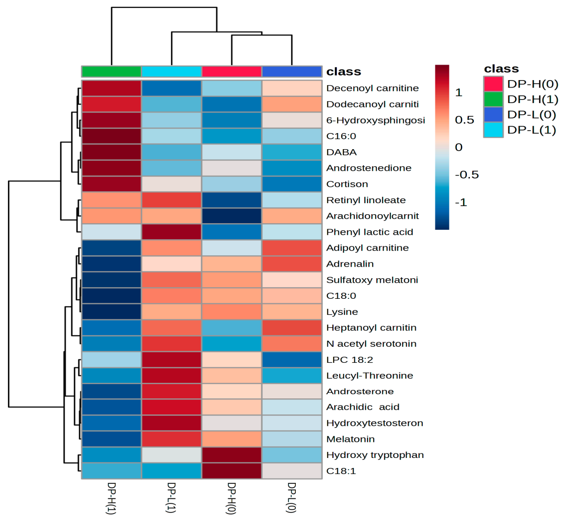


(b)

RF-graph (a) and Heatmap (b) illustrating the most representative molecules which may discriminate the DP-H *vs* DP-L burnout scores of day (0) *vs* night work (1) subjects.

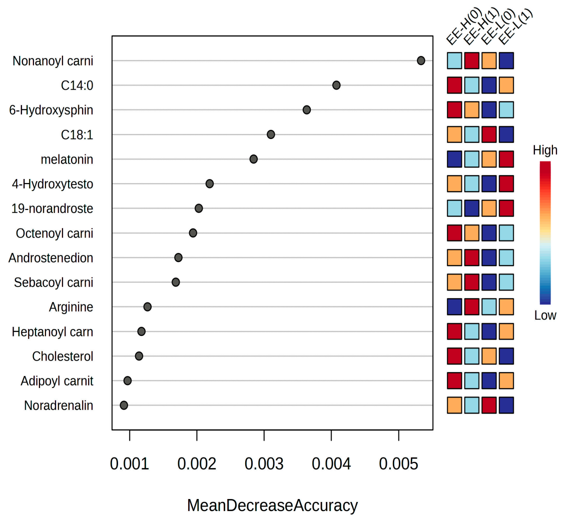


(a)

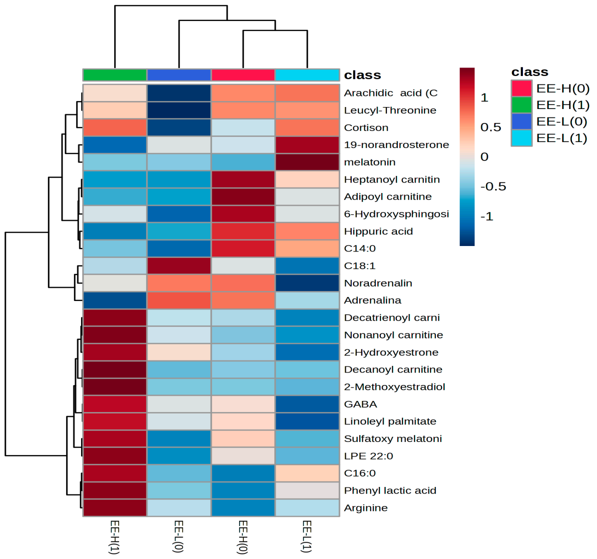


(b)

RF-graph (a) and Heatmap (b) illustrating the most representative molecules which may discriminate the EE-H *vs* EE-L burnout scores of day (0) *vs* night work (1) subjects.

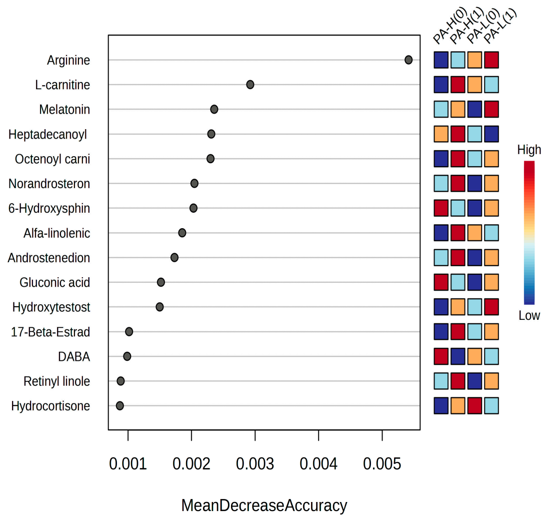


(a)

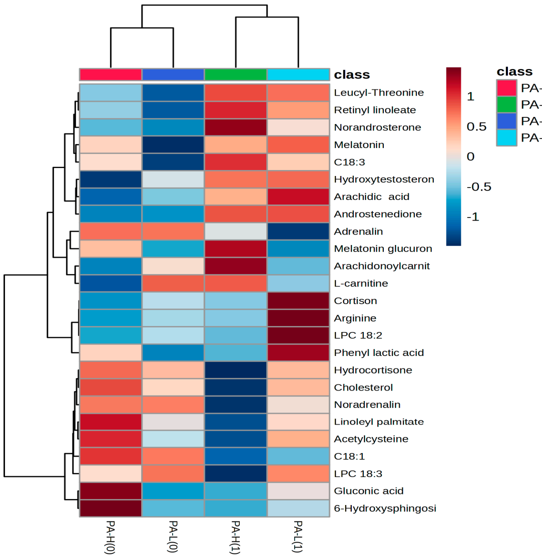


(b)

RF-graph (a) and Heatmap (b) illustrating the most representative molecules which may discriminate the night work subjects having PA-H *vs* PA-L burnout scores of day (0) *vs* night work (1) subjects..
