# Supplementary material for: Urine Metabolomic Patterns to Discriminate the Burnout Levels and Night-Shift-Related Stress in Healthcare Professionals
Source: Metabolites. 2025 Apr 14;15(4):273. doi: 10.3390/metabo15040273 (PMC12029983; doi:10.3390/metabo15040273)
Supplement: Supplementary file 1 [file metabolites-15-00273-s001.zip › Table S1 urine Identification class categ.pdf]

Supplementary file S1

Table S1. Urine molecules (n=79) separated using HPLC-QTOF-ESI+-MS and identified, according to their m/z values. The experimental m/z values were compared with the average of theoretical m/z values from the International database HMDB (Human Metabolomic DataBase). The accuracy of (theoretical – experimental) m/z values was below 20 ppm. The ID HMDB codes are mentioned in column 3.

m/z	Identification	ID (HMDB)
**TCA metabolites (n=5)**		
105.0348	Gama-amonobutyric acid (GABA)	HMDB0000112
119.0869	Diamino butyric acid (DABA)	HMDB0006284
130.0658	Pyroglutamic acid	HMDB0304793
151.1152	Phenyl lactic acid	HMDB0000814
197.1196	Gluconic acid	HMDB0000625
**Amino acids, amines and neurotransmitters (n=18)**		
134.0615	Asparagine	HMDB0000168
147.0824	Lysine	HMDB0000182
164.0404	Acetylcysteine	HMDB0001890
170.0669	Noradrenalin	HMDB0000216
175.1341	Arginine	HMDB0000517
180.1390	Hippuric acid	HMDB0000714
184.1005	Adrenalin	HMDB0000068
189.0506	Azelaic acid	HMDB0000784
205.1980	Tryptophan	HMDB0000929
209.1580	Kynurenine	HMDB0000684
221.1214	5 Hydroxy tryptophan	HMDB0000471
233.1580	Leucyl-Threonine	HMDB0028939
233.2310	Melatonin	HMDB0001389
275.1671	Serotonine sulfate	HMDB0240717
298.3553	N acetyl serotonin sulfate	HMDB0001238
329.2380	Sulfatoxy melatonin	HMDB0041815
394.3654	N acetyl serotonin glucuronide	HMDB60833
409.2682	Melatonin glucuronide	HMDB0060830
**Lipids: Fatty acids, sphingolipids and ceramides (n=15)**		
229.146	Myristic acid C14:0	HMDB0000806
255.0007	Palmitoleic acid (C16:1)	HMDB60082
257.2524	Palmitic acid (C16:0)	HMDB0000220
279.1657	Alfa-linolenic acid	HMDB0001388
279.2384	Linolenic acid (C18:3)	HMDB0003073
281.2544	Linoleic acid (C18:2)	HMDB0000673
283.2309	Oleic acid (C18:1)	HMDB0000207
285.2967	Stearic acid (C18:0)	HMDB0000827
307.2551	Eicosatrienoic acid (C20:3)	HMDB0002925
311.1926	Eicosenoic acid (C20:1)	HMDB0002231
313.2426	Arachidic acid (C20:0)	HMDB0002212
316.3296	6-hydroxysphingosine	LMSP01080003
505.3556	Linoleyl palmitate	LMFA07010126
540.4376	Ceramide(d18:0/16:0)	HMDB0011760
547.2044	all-trans-retinyl linoleate	LMPR01090016
**AcylCarnitines (n=14)**		

162.0574	L-carnitine	HMDB0000062
274.2804	Heptanoyl carnitine (C7:0)	HMDB0013129
286.3174	Octenoyl carnitine (C8:1)	HMDB0013324
290.2761	Adipoyl carnitine (C6:1; O2)	HMDB0061677
302.2038	Nonanoyl carnitine (C9:0)	HMDB0013288
310.2095	Decatrienoyl carnitine (C10:3)	HMDB0013325
314.2406	Decenoyl carnitine (C10:1)	HMDB0241072
316.2187	Decanoyl carnitine (C10:0)	HMDB0000651
344.2168	Dodecanoyl carnitine (C12:0)	HMDB0000944
346.2317	Sebacoyl carnitine (C10:1;O2)	HMDB0240726
400.3913	Palmitoyl carnitine	HMDB0240952
414.3365	Heptadecanoyl carnitine	HMDB0006210
424.3764	Linoleoyl carnitine (C18:2)	HMDB0006469
448.3221	O-arachidonoylcarnitine (C20:4)	HMDB0006460
**Steroids and vitamins (n=23)**		
275.2638	alpha-androstenol	HMDB0005935
277.1839	19-norandrosterone	HMDB0002697
287.1727	2-Hydroxyestrone	HMDB0000343
287.1864	Androstenedione	HMDB0000053
289.1413	Estriol	HMDB0000151
289.1617	Testosterone	HMDB0000234
291.2598	Androsterone	HMDB0000031
301.1493	2-Methoxyestrone	HMDB0000010
303.2394	2-Methoxyestradiol-17beta	HMDB0000405
303.2606	17-Methyltestosterone	HMDB0015655
305.1787	4-Hydroxytestosterone	HMDB0246468
361.2349	Cortisone	HMDB0002802
363.2248	Cortisol	HMDB0000063
363.2316	Hydrocortisone	HMDB0002802
365.2885	Tetrahydrocortisone	HMDB0000903
365.1479	Dihydrocortisol	HMDB0003259
369.2536	Dehydroepiandrosterone 3-sulfate (DHAS)	HMDB0001032
387.209	Cholesterol	HMDB00030
397.3046	Ergocalciferol (Vit D2)	HMDB0000900
401.2792	5,6-trans-25-Hydroxyvitamin D3	HMDB0006721
433.2723	17-Beta-Estradiol-sulfate	HMDB0041620
539.2353	Hydrocortisone glucuronide	HMDB0010359
653.6169	18:0 Cholesterol ester	HMDB0010368
**Phospholipids (n=4)**		
518.3183	LPC 18:3	HMDB0010387
520.3557	LPC 18:2	HMDB0010386
539.3818	LPE 22:0	HMDB11520
782.6044	PC(18:2/18:2)	HMDB0008138
